# Time perception of attractive male faces and voices: The role of women’s menstrual cycle

**DOI:** 10.1371/journal.pone.0321956

**Published:** 2025-04-24

**Authors:** Nuno Fernandes, Sandra Soares, Mavilde Arantes, Joana Arantes

**Affiliations:** 1 School of Psychology, University of Minho, Braga, Portugal; 2 Department of Education and Psychology, University of Aveiro, Aveiro, Portugal; 3 Department of Imaging, Portuguese Institute of Oncology, Porto, Portugal; 4 Unit of Anatomy, Department of Biomedicine, Faculty of Medicine, University of Porto, Porto, Portugal; Hangzhou Normal University, CHINA

## Abstract

Accurate time measurement is essential for organisms to synchronize their internal biological cycles with the external environment, crucial for reproductive success and survival. This study examines fluctuations in time perception across different phases of the menstrual cycle in response to visual and vocal attractiveness, building on previous research showing that women estimate longer viewing times for attractive male photos compared to unattractive ones. Forty-eight females participated in the experiment during both the menstruation and ovulation phases of their menstrual cycles, completing visual and auditory oddball tasks. Participants viewed a series of five stimuli and reproduced the duration of the last stimulus by pressing a mouse button. The final stimulus could be identical to the previous four (control trials), an attractive or unattractive male photo (visual attractiveness block), a masculinized or feminized male face (visual sexually dimorphic block), or a masculinized or feminized male voice (auditory sexually dimorphic block). Results indicated that duration estimates for masculinized male voices were significantly longer compared to feminized voices, while no differences were found for the menstruation phase. However, no differences were observed between the phases of the menstrual cycle in the visual conditions. Nevertheless, we replicate the temporal dilation effect associated with viewing attractive stimuli, suggesting an acceleration of the internal clock related to attractiveness perception. Our findings align with the literature on this phenomenon and provide initial evidence for an adaptive time perception mechanism influenced by the menstrual cycle, contributing to the understanding of the complex interactions between biological cycles and perceptual processes.

## Introduction

The accurate measurement of time allows organisms to align their internal biological cycles with the external environment, which is crucial for reproductive and survival purposes [1]. Yet, pop culture often describes feelings of time freezing when people are in love or attracted to others. But do these assertions find scientific support? Previous research has indicated that women’s estimated viewing time of attractive male photos was longer than corresponding estimates for unattractive ones [2–4]. Does the perception of time fluctuate across different phases of the menstrual cycle, similar to how judgments of attractiveness do [5,6]. Additionally, to what extent can these results be extrapolated to other sensory domains?

### The human experience of time

A model for the “internal clock” based on a pacemaker, counter, store, and comparator was initially proposed to provide explanations for many findings concerning time perception [7], such as the notion of Weber’s law, which suggests that stimulus sensitivity will increase proportionally with increases in stimulus intensity [8]. Later, the Scalar Expectancy Theory (SET; [9]) was formulated, which remains the most influential pacemaker-accumulator model of time perception. According to SET, pulses are generated by an arousal-related pacemaker and counted by an accumulator. These counts are stored in memory when an attention-controlled switch closes. For temporal judgments, a comparator assesses the accumulator’s current count against values from previous exposures to the timed interval, determining whether the duration is similar. Many factors influence the perception of time, such as attractiveness [2–4,10,11].

### Time perception of attractive photos

Recent studies have investigated the duration estimates of viewing attractive opposite-sex photos [2,4]. Researchers showed that females’ duration estimates of attractive male photos were significantly longer than corresponding estimates for unattractive male images [2]. Later, it was found that, for both sexes, the duration estimates of attractive opposite-sex faces were longer than for unattractive opposite-sex faces [4]. Also, a study using a speed dating methodology found that when there is a holistic perception of the partner as physically more attractive, time seems to slow down for women [3].

Moreover, other researchers showed a female overestimation of same-sex attractive photos compared to unattractive ones, and that angry facial expressions were overestimated, but only for attractive faces [12]. Similar to the suggested mechanism by which emotional arousal stimuli, like angry faces [13–15], or auditory stimuli, such as unpleasant music [16], are believed to impact time perception by elevating the pacemaker rate [13], the same mechanism is thought to come into play when viewing attractive stimuli [2]. Additionally, the role of arousal in the effect of attractiveness on time perception was empirically tested, showing that increased arousal mediated the temporal dilation effect of attractive faces [11]. However, that study included only male participants.

Alternative explanations for the phenomenon may also be considered. For instance, it was observed that female participants underestimated the duration of same-sex unattractive faces in comparison to attractive and neutral faces [10]. The author attributed these findings to attention being diverted from time perception, as supported by the Attention-Gate Model (AGM; [17,18]). The AGM suggests the use of an attentional gate instead of the switch observed in the SET. In contrast to the SET switch, presumed to remain closed once activated during a specific timing instance, the AGM gate can open and close intermittently throughout the timing process, being influenced by the level of attention allocated to the timing task. Despite the study exclusively employing same-sex photos [10], it is essential not to disregard the impact of attention on the perception of time when exposed to attractive stimuli. Alongside arousal, attention emerges as one of the most influential determinants of time perception [19].

### The impact of menstrual cycle dynamics on attractiveness perception

For women, seeing an attractive man is likely to engage the appetitive motivational system by eliciting physiological reflexes that mobilize the organism for action (for a review, see [20]). Accordingly, there is substantial evidence that viewing photos of attractive opposite-sex individuals elicits automatic or implicit cognitive processes. For instance, it was found that viewing erotic photographs elicited autonomic physiological responses (e.g., increased skin conductance, cardiac deceleration, and startle reflex modulation) [21]. Additionally, it was observed that women’s autonomic arousal (pupils dilated) was higher when viewing photos of their boyfriends and famous male actors compared to pictures of female actresses or other participants’ boyfriends [22]. Interestingly, this effect was increased during the ovulatory phase of the menstrual cycle and not obtained for women who were taking oral contraceptives, suggesting an influence of the menstrual cycle hormones on these physiological responses. Furthermore, using a GO/NOGO paradigm with functional MRI, evidence for changes in women’s brain activity across the menstrual cycle was found when processing attractive male faces [23]. The study identified instances of both successful inhibitions and commission errors that were exclusive to the male stimuli (attractive female photos were also used) during the fertile phase of the menstrual cycle.

The menstrual cycle comprises different hormonal arrangements [24]. Precisely, during the late follicular period, a peak of estradiol is followed by a rise in progesterone. Consequently, females experience several motivational [25,26], perceptual [27,28], and cognitive [29,30] changes. On the realm of mating preferences, although findings are mixed, there is evidence supporting the good genes ovulatory shift hypothesis (for a review, see [31,32]). This hypothesis suggests that in the fertile phase of the cycle, women exhibit stronger preferences for men with masculine traits compared to when they are in their lowest-fertile phase. Additionally, it is theorized that when women are at a lower risk of conception, they tend to prefer a less masculine partner who may be more likely to provide resources for offspring [32]. A meta-analysis conducted by [33] supported these claims, revealing that women showed a shift in preferences during their menstrual cycle when assessing potential short-term partners (e.g., for a one-night stand) or when assessing the attractiveness of male stimuli without specifying a relationship context, but not when evaluating male stimuli as potential long-term partners (e.g., for marriage).

Despite mixed findings, masculinity in men is generally believed to signal health and “good genes” (i.e., a better immune system) [32,34–37]). This is based on the idea that masculinity indicates higher basal testosterone levels [38,39]. Therefore, a key rationale for predicting hormone-linked changes in women’s preferences for facial masculinity is the assertion that facial masculinity reflects men’s heritable immunocompetence [40]. Although exploring the underlying motives for sexually dimorphic preferences among women is not the focus of the present work, there is evidence challenging the immunocompetence handicap hypothesis (intersexual competition) [41–44]. Instead of serving as an indicator of men’s immunocompetence, men’s masculinity may primarily signal their intrasexual competitiveness (for a review, see [45,46]).

Additionally, menstrual cycle shift preferences are also observed for other sensory modalities. Men with higher testosterone levels speak with lower fundamental frequencies (F0) and are judged as being more masculine [47], attractive [48,49], and have higher mating success [50]. Also, researchers found that masculine voices are associated with better disease resistance, contrasting with the immunocompetence hypothesis predictions [51,52]. However, the authors warn that further studies, including testosterone level manipulation, are necessary to determine whether androgens should be treated as immunomodulators rather than implicit immunosuppressants, as suggested by [53].

Despite the theoretical framework employed, there is evidence suggesting that the preference for masculine voices is heightened during the late follicular (fertile) phase of the menstrual cycle compared to the early follicular and luteal (non-fertile) phases [54]. In a subsequent study, using estimated hormonal measures based on the menstrual cycle, researchers found that preferences for vocal masculinity in naturally cycling women decreased with higher predicted progesterone levels and increased with higher predicted prolactin levels [55]. This pattern did not appear in women using hormonal contraceptives. Moreover, other researchers corroborated these findings, revealing that women’s preference for masculine voices was strongest when estradiol levels were high [56].

Extending the effect of attractiveness on time perception to auditory stimuli is particularly relevant given the disparities in the literature regarding preferences for masculinity in faces and voices. For instance, a recent meta-analysis found no clear evidence that facial masculinity predicts mating success, whereas male voice pitch and testosterone levels were significant predictors [57]. Additionally, time judgment differs between auditory and visual stimuli, with auditory stimuli generally perceived as longer than visual stimuli of comparable duration (for a review, see [58]). Although research demonstrated that women exhibited decreased accuracy in discriminating tone durations during the premenstrual phase [59], no previous studies have explored women’s estimates of the duration of masculine voices across different phases of the menstrual cycle.

Despite the rationale of the good genes ovulatory shift hypothesis presented in this work, there is an ongoing debate in the literature about whether women’s hormonal status influences their preferences for masculinity. In contrast to previous findings [33], another meta-analysis found no evidence that women in their fertile phase exhibit a stronger preference for short-term relationships with men presumed to have high genetic quality (i.e., high testosterone, masculinity, dominance) [60]. The authors attribute these conflicting findings to research that used broader, less precise methods of determining the fertile phase.

For instance, other research failed to find support for the hypothesis that women are attracted to men with masculine faces when conception is likely but prefer men with feminine faces during other phases of the menstrual cycle [61,62]. Despite some criticism towards [61,62], primarily concerning the small sample size and the use of counting methods to estimate fertility [63,64], other researchers have failed to support this effect [65–67]. Moreover, the largest longitudinal study carried out to date on the hormonal correlates of women’s preferences for facial masculinity (*n* = 584) did not find significant evidence that changes in women’s salivary steroid hormone levels were related to their preferences for facial masculinity [65]. Additionally, a large within-subject study (*n* = 257), which included salivary hormone measurements and luteinizing hormone tests, found no evidence of shifts in attraction to men’s bodies throughout the menstrual cycle [67]. There is also mixed evidence regarding preferences for masculinity in voices throughout the menstrual cycle. For instance, no evidence was found that cycle phase, conception risk, or steroid hormone levels influence women’s preferences for masculine voices [68].

Overall, despite the mixed findings in the literature, there are reasons to believe that these discrepancies could be attributed to the method of hormonal measurement, the types of stimuli used, or both. For instance, preferences for facial masculinity during the fertile phase were not captured using LH tests [69]. However, consistent with the ovulatory shift hypothesis, rising estradiol (linked to increased fecundability) predicted a preference for more masculine faces, while high progesterone (linked to decreased fecundability) predicted a preference for more feminine faces [69]. Additionally, ovulatory shifts in women’s preferences for masculinity in men were also observed in a speed-dating scenario, by estimating the probability of conception [70]. This presents evidence against the notion that, if the effect exists, it would be too small to appear outside of a carefully controlled scenario using artificial stimuli. Therefore, during ovulation, one might expect a slowing down of subjective time (i.e., increased subjective duration) when in the presence of an attractive mate to collect and process as much information as possible about the potential partner.

### The present study

Accordingly, the present study aims to test whether females’ duration estimates of brief exposures to masculine faces and voices change during different phases of the menstrual cycle (fertile vs. non-fertile phase). In the fertile phase of the menstrual cycle, when women unexpectedly meet a masculine man, increased arousal levels should lead to longer perceived durations of that encounter. Therefore, our first hypothesis (H1) is that during the fertile phase of the cycle, women will judge the duration of briefly viewed attractive male photos to be longer than unattractive male photos, but not during the non-fertile phase. In addition, we expect that during the fertile phase, women will reproduce the recently viewed masculinized male photos longer than feminized male photos (H2). Finally, we predict that in the fertile phase of the cycle, the same effect will occur when exposed to auditory stimuli (i.e., longer perceived durations of briefly heard masculinized male voices than femininized male voices) but not during the non-fertile phase (H3).

## Method

### Participants

We recruited a total of 78 female participants from 15 February 2022 to 15 January 2023. Of these, 14 attended the screening session but dropped out before the first experimental session. An additional nine participants dropped out after completing the menstruation phase, and five more dropped out after the ovulation phase. Reasons for dropout included difficulties obtaining a positive ovulation test (e.g., anovulatory cycle, unexpected cycle length variations) and participants’ inability to attend the lab during specific periods (e.g., weekends, holidays, exams). This dropout rate (36%) is consistent with previous studies using similar methods (e.g., 42% in [71], 39% in [72], and 28% in [73]). Of the 50 participants who completed both phases of the experiment, two were excluded from the analysis because they did not meet the inclusion criteria for sexual orientation.

Therefore, our final sample consisted of 48 female participants aged 18 to 25 years (*M*_*age*_ = 20.78; *SD* = 1.90). This sample size is consistent with that of previous studies [2,4,10,12]. We lack data for the auditory block of the menstrual phase for participant "VA606" due to technical issues during the experiment. A posteriori power analysis using simr [74], for 1000 simulations, revealed that our study had 80.40% power to detect the triple interaction term of trial type*block type*session. The participants met the following criteria: they were heterosexual or bisexual, were not using hormonal contraception (and had not been using hormonal contraceptives for at least one month), reported having a regular menstrual cycle (*M*_*days*_ = 29.3; *SD* = 2.5), and reported no hearing or visual uncorrected problems. Detailed characteristics of the participants are publicly available [75]. The main characteristics can also be consulted in S1 Table. Participants were recruited through institutional email and online social networks (e.g., Facebook), and each received a 30-euro voucher as compensation for their participation. Psychology students (*n* = 22) were granted course credits for their participation in addition to receiving the voucher (post-hoc analysis suggests no influence on the results).

### Apparatus and stimulus

The experiment was conducted on an IBM-compatible PC, and responses were recorded using the mouse. The stimuli were presented on a gray background in the center of a 17″ LCD screen (1,920 × 1,080 pixels, 60 Hz). The experimental task was programmed in PsychoPy (v2022.1) [76].

For the visual blocks, the neutral stimuli were circular Gabor discs, which are sinusoidal gratings modified by a Gaussian filter [77]. In the present study, Gabor discs were identical, measuring 5 cm in diameter, oriented 10° clockwise from the vertical axis, and with a 3 cycles/disc frequency. The test stimuli comprised either color photos of attractive and unattractive adult males (attractiveness condition) or masculinized or feminized versions of adult male faces (sexually dimorphic condition).

The visual attractiveness block consisted of five attractive and five unattractive head and head-and-torso male photos, which had already been employed in previous research [2]. The visual sexually dimorphic set consisted of five individual male faces with neutral expressions that were masculinized and feminized using Psychomorph’s (version 6) prototype-based computer graphics transformations [78]. Photos for both conditions were resized to 500 x 500 pixels and were displayed in the center of the screen at the same size as the Gabor discs. The photos used in the sexual dimorphism manipulation were selected from the Face Research Lab London Set [79]. The female and male prototypes were created by averaging the shape, color, and texture of 30 female and 30 male faces. Then, masculinized and feminized versions were manufactured by adding or subtracting 50% of the linear differences between female and male prototypes to (or from) each male photo (see [80] for technical detail). An example of a feminized version can be found in S1 File, and a masculinized version in S2 File.

All photos were assessed by a sample of 10 female raters (*M*_age_ = 21.9; *SD* = 3.24), who were asked to rate the attractiveness of each photo on a 10-point scale (1 = extremely unattractive; 10 = extremely attractive). The photos were presented in one of two random orders. The average rating of the attractive images was 8.32, while the unattractive photos received an average rating of 1.54, *t*(9) = 28.03, *p* <.001. The average attractiveness rating for the masculinized faces was 4.68, whereas, for the feminized faces, it was 3.70, *t*(9) = 2.82, *p* <.01. During debriefing, none of *t*he raters or participants reported having recognized any of the men in the photos. Moreover, none of the participants involved in the validation process participated in the study.

For the auditory block, five men’s voices were recorded, uttering the monophthong vowel/i/ with an iPhone 14 Pro in a soundproof booth from a distance of approximately 20 cm. To ensure the neutrality of the men’s voices (neither masculine nor feminine), six female raters (*M*_age_ = 21.20; *SD* = 2.36) were asked to rate 10 male voices in terms of masculinity on a 10-point scale (1 = extremely feminine; 10 = extremely masculine). The voices were presented in a random order. The five voices classified as the most neutral were retained (*M* = 5.24; *SD* = 1.91). Upon debriefing, none of the raters or participants reported recognizing any voices. The voices were directly encoded onto a computer hard disk in mono at a 48.0 kHz sampling rate and 32-bit quantization using Audacity [81]. All acoustic manipulations were performed with the Praat Software version 6.1.50 [82].

Firstly, voices were compressed or stretched to values between 0.133 s and 2.1 s. Subsequently, voices were transformed by raising or lowering the fundamental frequencies (pitch) [48]. The fundamental frequencies were reduced by 20 Hz to masculinize and raised by 20 Hz to feminize them. Finally, the amplitude of each sound was normalized to 70 dB. These manipulated voices were then presented to seven female raters (*M =* 21.03*; SD =* 2.23*),* who were asked to rate the voices for attractiveness on a 10-point scale (1 = extremely unattractive; 10 = extremely attractive). The voices were presented in a random order. The average rating of the masculinized voices was 5.80, while the average rating for the feminized voices was 4.17, *t*(6) = 3.48, *p* <.01. Upon debriefing, none of the raters or participants reported having recognized any of the male voices. Furthermore, none of the participants involved in the validation process participated in the study.

### Ethics

All participants gave their written informed consent, according to the Helsinki Declaration. This study was approved by the Ethics Committee for Research in Social and Human Sciences of the University of Minho (CEICSH 056/2021).

### Procedure

Before the experimental sessions, participants completed questions related to the inclusion criteria and received instructions regarding the experimental procedure and the ClearBlue© Digital Ovulation Test. These tests measure luteinizing hormone (LH) in urine, which rises approximately 24–48 hours before ovulation [83]. The manufacturer, Swiss Precision Diagnostics, asserts that this product can identify the two most fertile days with an accuracy rate of over 99%. This claim is supported by independent research showing that LH tests are 97% accurate in confirming ovulation as detected by ultrasound [84]. Subsequently, participants performed the experimental task during their menstruation and ovulation phases of the menstrual cycle. For the menstruation phase, participants came to the within 3 days after the onset of menses. Regarding the ovulation phase, participants took an ovulation test every morning on specific days.

The calculation for the first day’s test involved dividing the average length of the participant’s menstrual cycle by two (indicating theoretical ovulation occurrence) and then subtracting 5 days from that result. Upon receiving a positive result on the test, participants came to the lab to perform the experimental task corresponding to the ovulation phase. If they were unable to come to the lab within the following 48 hours after the positive result (when the chances of pregnancy were highest), the ovulation experimental task would be postponed until the next ovulation cycle. Out of the 48 participants, 28 began the experiment during the menstruation phase, while 20 started during the ovulation phase.

For the experimental task, participants were tested in individual sound-isolated booths and seated approximately 60 cm away from the monitor while wearing headphones. They were informed that they would see or hear sequences of five stimuli and were instructed to reproduce the duration of the fifth stimulus in each sequence by pressing the left mouse button for as long as they thought the stimulus was presented to them. After the duration reproduction of the fifth stimulus, the following trial would start precisely 2 seconds (s) later. Before beginning the experimental trials, participants were required to respond as accurately as possible and to complete three practice trials. These practice trials involved stimuli that were either Gabor discs or neutral auditory stimuli, depending on the block.

The experimental task consisted of three test blocks, each consisting of 105 trials, with a 5-minute interval between blocks in which participants engaged in a filler task. In each test block, either attractive/unattractive male photos or masculinized/feminized male photos or voices were used as test stimuli. The order of the blocks was counterbalanced across participants. In the visual stimuli blocks, in each trial four Gabor discs were displayed for a similar duration, separated by an inter-stimulus interval (ISI), and followed by a test stimulus. In one-third of the trials, the test stimulus was either a Gabor disc (neutral condition), an attractive/masculinized male photo (attractiveness/sexual dimorphism conditions), or an unattractive/feminized male photo (attractiveness/sexual dimorphism condition). In the auditory stimuli block, four 600 Hz sine wave sounds were played for a similar duration, separated by an ISI and followed by a test stimulus. In one-third of the trials, the test stimuli were either the 600 Hz sine wave sound (neutral condition), a masculinized male voice (masculinized condition), or a feminized male voice (femininized condition).

**Fig 1:**
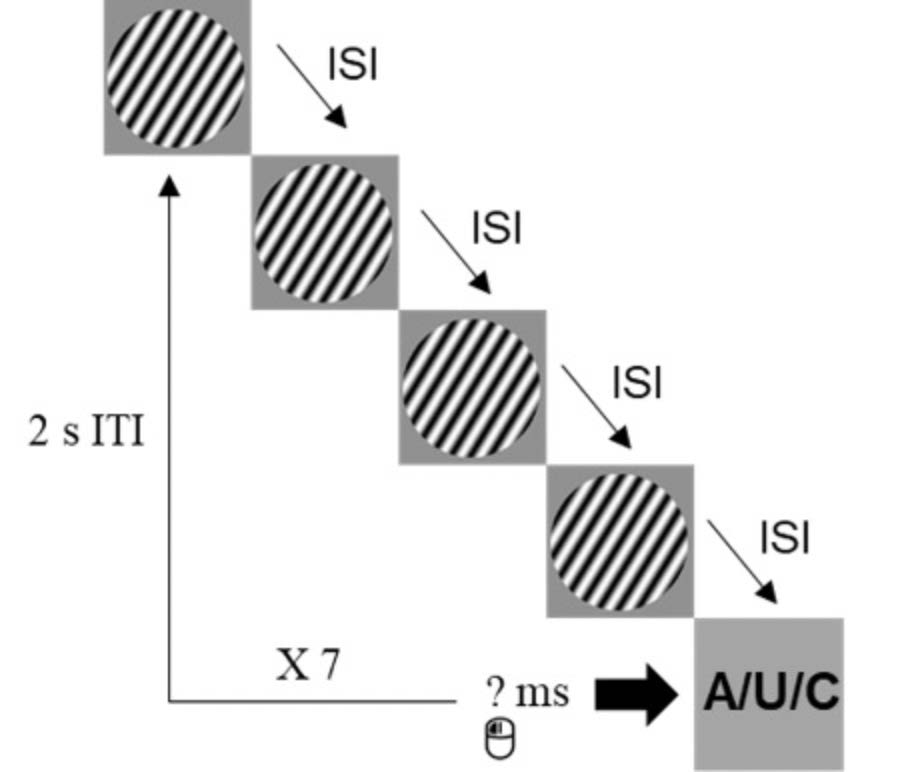
The oddball task for the visual attractiveness condition. A – attractive male photo; U – unattractive male photo; C – control (Gabor disc); ISI – inter-stimulus interval; ITI – inter-trial interval; ms – milliseconds.

Similar to previous research [2], we used seven possible stimulus durations in millisecond intervals: 133 ms, 233 ms, 300 ms, 383 ms, 533 ms, 1050 ms, and 2100 ms, each occurring on an equal number of times (pseudorandomly distributed). Moreover, each of the three conditions (neutral, attractive/masculinized, unattractive/feminized) had five replications at each duration per block. The five attractive/masculinized and unattractive/feminized stimuli were presented once at each duration. The ISIs within a trial varied by +/- 10% of the stimuli duration to ensure that participants would respond to the stimuli duration and not a beat or rhythm that might occur if the ISI was kept constant throughout the trial [85]. The experimental task for the visual attractiveness condition is illustrated in Fig 1.

### Data analysis

Since the reproduced durations were right-skewed, a logarithmic transformation was applied to normalize the distribution. The log-transformed reproduced duration was then entered as the dependent variable in a linear mixed-effects model (LMM) with by-subject random intercepts. The triple interaction term of block (visual attractiveness vs. visual dimorphic vs. auditory dimorphic), trial type (attractive/masculinized vs. unattractive/feminized vs. control), and phase of the cycle (menstruation vs. ovulation), as well as the three main effects, were included as fixed terms. Multilevel modeling was used to account for the nesting of the choice data. We also controlled for the order of the menstrual cycle phase in which the participant completed the experiment and their relationship status, but these factors did not influence our results.

In a preprocessing stage, following methods of outlier detection [86], trials that were larger than the third quartile plus 1.5 times the Interquartile range (IQR) (>_*q*0.75_ + 1.5×IQR) or smaller than the first quartile minus 1.5 times the IQR (< _*q*0.25_−1.5×IQR) were removed from the analysis (5% of the visual attractiveness and auditory dimorphic block; and 7% of the visual dimorphic block), to increase the signal to noise ratio. However, results regarding statistical comparisons were unchanged if these trials were included. Statistical analysis was performed using R [87] in RStudio Version 2022.2.2.485 [88]. The data and reproducible code are publicly available [75].

## Results

### Vierordt’s Law

**Fig 2:**
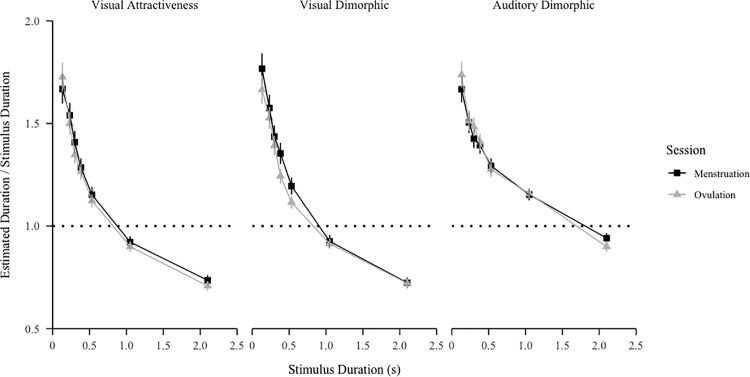
The ratio of estimated duration to stimulus duration for each block averaged across test and control trials. Error bars represent the standard error of the mean.

We initially assessed whether reproduced durations in both phases of the cycle conformed to Vierdodt’s Law [89], which states that shorter intervals are typically overestimated, while longer intervals are underestimated (for a review, see [90]). For each phase (ovulation/menstruation) and block (visual attractiveness/visual dimorphic/auditory dimorphic), we calculated the ratio of each estimated duration to the objective duration and averaged these ratios over condition (neutral/attractive/unattractive) for each participant. Results are shown in Fig 2. In the visual blocks (attractiveness and dimorphic), durations were overestimated for stimuli shorter than 1.05 s and underestimated for stimuli lasting 1.05 s or 2.1 s in both phases, aligning with expectations based on Vierordt’s Law. In the auditory dimorphic block, durations were overestimated for stimuli shorter than 2.1 s and underestimated for stimuli lasting 2.1 s for both phases, which also conforms to Vierordt’s Law, and is consistent with previous findings reporting longer perceived durations of auditory stimuli (e.g., [91–93]).

### Linear mixed-effects model (LMM)

The Linear mixed-effects model (LMM) included the main effects of block type, trial type, menstrual cycle phase, and their three-way interaction term, as fixed terms. Also, we controlled for trial duration, by including this factor as a fixed term in the model. The log-transformed time reproduction was entered as the dependent variable. Participant code was entered as a random intercept. The analysis revealed that 56.9% of the variance (R^2^_marginal_ = 0.569) could be attributed to the fixed effects alone. The R^2^_conditional_ was found to be 0.701, suggesting that 70.1% of the variance is explained by the combined fixed and random effects.

The model showed a significant main effect for trial type, *F*(2, 28348) = 39.53, *p* <.001, for block, *F*(2, 28349) = 286.59, *p* <.001, and for trial duration, *F*(7, 28348) = 7609.74, *p* <.001. Additionally, the two-way interaction between trial type and block was statistically significant, *F*(4, 28348) = 24.37, *p* <.001. The main effect of session was not statistically significant, *F*(1, 28352) = 0.64, *p* =.42. Also, neither the interaction of session and trial type, *F*(2, 28348) = 2.40, *p* =.09, nor the interaction of session and block, *F*(2, 28349) = 2.19, *p* =.11, However, the three-way interaction of session, trial type and block showed significant results, *F*(4, 28355) = 3.09, *p* =.01.

**Fig 3:**
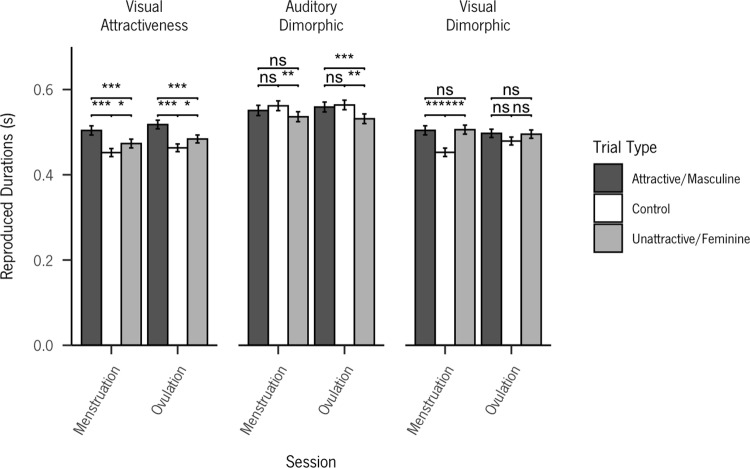
Average reproduced durations by menstrual and ovulation phases across all conditions. Error bars represent the standard error of the mean.

Since a significant three-way interaction was found between session, trial type, and block, post-hoc comparisons using the Tukey adjustment were performed (Table 1). Post-hocs revealed that the influence of the cycle phase was only observed for the auditory sexually dimorphic block, with masculinized voices being perceived for longer than feminized ones exclusively during ovulation (*p* <.001). For the visual attractiveness block, attractive faces were perceived as longer than unattractive ones, both for the ovulation (*p* <.001) and menstruation phase (*p* <.001). No differences were found for the visual sexually dimorphic block. The average reproduction durations across all conditions in both phases of the menstrual cycle are illustrated in Fig 3. Also, visualizations of time reproductions for each of the seven durations across the various conditions are provided in S1–S3 Figs.

**Table 1:** Tukey HSD comparison for Block Type*Trial Type.

Session	Block Type	(I) Trial Type	(J) Trial Type	% Diff(I-J)^a^^,^^b^	Std. Error	*df* ^c^	*t*	95% Confidence Interval	
								Lower Bound	Upper Bound
Ovulation									
	Visual Attractiveness	attractive	control	11.6***	0.02	28348	7.12	0.18	0.32
		attractive	unattractive	7.3***	0.02	28348	4.48	0.09	0.23
		control	unattractive	-3.9*	0.02	28348	-2.64	-0.16	-0.02
	Visual Dimorphic	masculine	control	4.1	0.02	28348	2.33	0.01	0.15
		masculine	feminine	1.1	0.02	28348	0.28	-0.06	0.08
		control	feminine	-2.96	0.02	28348	-2.04	-0.14	-0.01
	Auditory Dimorphic	masculine	control	1.1	0.02	28348	0.15	-0.06	0.07
		masculine	feminine	6.2***	0.02	28348	3.65	0.06	0.20
		control	feminine	6.2**	0.02	28348	3.51	0.05	0.19
Menstruation									
	Visual Attractiveness	attractive	control	11.6***	0.02	28348	6.79	0.17	0.31
		attractive	unattractive	6.2***	0.02	28348	3.88	0.07	0.21
		control	unattractive	-4.9*	0.02	28348	-2.91	-0.17	-0.03
	Visual Dimorphic	masculine	control	11.6***	0.02	28348	6.74	0.17	0.31
		masculine	feminine	-1.0	0.02	28348	-0.12	-0.7	0.07
		control	feminine	-10.4***	0.02	28348	-6.85	-0.32	-0.18
	Auditory Dimorphic	masculine	control	-2.96	0.02	28348	-1.58	-0.13	0.01
		masculine	feminine	3.05	0.02	28348	1.68	-0.01	0.13
		control	feminine	5.13**	0.02	28348	3.25	0.05	0.19

^a^* *p* <.05, ** *p* <.01, *** *p* <.001.

^b^Represents the percentage change, obtained by exponentiating the log-difference and converting it to percentage.

^c^*df* calculated via Satterthwaite method.

## Discussion

The current study aimed to investigate whether females’ estimated duration of 1) briefly viewing opposite-sex attractive/unattractive faces and 2) briefly viewing and hearing opposite-sex sexually dimorphic (masculinized/feminized) faces and voices would depend on the menstrual cycle phase. Based on an evolutionary perspective, we expected that during the fertile phase of the cycle, attractive and masculinized stimuli would be perceived as longer than unattractive ones, but not during the non-fertile phase.

This prediction was tested in a visual and auditory oddball task in which participants viewed and heard series of five stimuli of equal duration and had to reproduce the duration of the last stimulus by pressing the mouse button. The first four stimuli on each trial were either circular sine-wave gratings (visual oddball) or 600 Hz sine waves (auditory oddball). The last stimulus could be either an identical stimulus (control condition), an attractive/unattractive male photo (attractiveness block), a masculinized/feminized male facial picture (visual sexually dimorphic block), or a feminized/masculinized male voice (auditory sexually dimorphic block).

We found evidence supporting a shift in time perception during the menstrual cycle regarding sexually dimorphic voices (H3). Specifically, we observed that masculinized male voices were reproduced for longer durations than feminized voices during ovulation, but this effect was not present during menstruation. However, we did not find evidence supporting a shift in the time perception of visual stimuli related to attractiveness across the menstrual cycle, neither in the attractiveness block (H1) nor in the sexually dimorphic block (H2). Nonetheless, we successfully replicated previous findings in the literature, demonstrating that briefly viewed attractive faces were reproduced for longer durations than unattractive ones.

### Visual attractiveness

Our first hypothesis posited that during the fertile phase of the cycle, women would perceive the duration of briefly viewed attractive male photos as longer than unattractive male photos, but not during the non-fertile phase. Our results replicate previous findings that support the role of arousal mechanisms in the temporal dilation effect of attractiveness [2–4,11] and contrast to a previous study suggesting a decrease in time perception for same-sex unattractive faces compared to attractive and neutral ones [10]. However, contrary to our hypothesis and suggestion from prior researchers [2], our data does not provide evidence to support the influence of the menstrual cycle on the effect of attractiveness on time perception.

The lack of a moderating role for the menstrual cycle could be attributed to the specific phases in which the participants were tested (ovulation and menstruation) or to the nature of the experimental task employed. In a study of electroencephalography (EEG) activity related to the perception of visual stimuli during different phases of the menstrual cycle [94], higher activation was found for both negative and positive emotional visual stimuli during the follicular phase, while minimum activation during ovulation was observed. Conversely, erotically colored visual stimuli induced the most intense changes in the EEG during the ovulatory phase. Surprisingly, these brain activity changes were restricted to the post-stimulus time interval and were not observed while viewing the pictures. Therefore, during our experiment, participants may have experienced heightened arousal in response to emotional stimuli during the menstruation phase, potentially explaining the observed longer perceived durations. Finally, since participants estimated the reproduced duration immediately after the stimulus onset, we may not have captured the prolonged and sustained effects of erotic stimuli during the ovulatory phase.

It is important to note that the literature does not clarify the hypothesis that changes in the visual perceptual system across the menstrual cycle are directly linked to patterns of fluctuating hormone levels. Previous research found that women tend toward higher values for a two-flash threshold task premenstrually, indicating decreased perceptual ability [95]. This result suggests lower arousal during the premenstrual phase of the cycle but not during the ovulation or the menstrual phase. Similarly, other research found an impaired performance in visual detection, both in threshold and sensitivity, during the premenstrual phase [96]. Additionally, they rejected the hypothesis that performance deterioration could be explained by mood changes associated with the menstrual cycle, pointing to physiological changes instead. Thus, these findings might provide alternative explanations for the lack of interaction between the effect of attractiveness on time perception and the menstrual cycle phase.

### Visual sexually dimorphic

Our hypothesis stated that during the fertile phase, women would perceive masculinized male photos as being presented for longer than feminized male photos. However, no differences were found between masculine and feminine male faces. In fact, although not statistically significant, the average reproductions for feminine faces were higher than those for masculine faces. Considering this, we would like to discuss the absence of evidence supporting differences in time reproduction between viewing masculine and feminine faces.

Although many studies have reported that women prefer masculine faces [48,97,98], some studies challenge these findings. For instance, prior work suggested a stronger preference for feminine male faces [99]. The authors found that enhancing masculinity in male faces increased perceived dominance and negative attributions (coldness or dishonesty). Further research revealed that under conditions of environmental harshness, such as scarcity of resources or high stress, women tend to prefer less masculine male faces for long-term relationships [100,101]. Additionally, a cross-cultural study showed that women’s preference for more masculine men is more robust in countries with higher economic and national health indexes [102], which also supports the idea that men with higher levels of testosterone are believed to invest less in relationships.

The notion that women’s preferences for facial masculinity are related to their hormonal status is also highly controversial in the literature. For example, neither [61] found evidence for a greater preference for masculine faces when fertilization was likely, nor support for a link between women’s preferences for facial masculinity and their hormonal status was found [65]. Similarly, a recent study using an eye-tracking paradigm failed to find shifts in women’s visual attention to facial masculinity across the menstrual cycle [103]. Researchers highlight that the absence of an effect could be attributed to either the use of hormonal contraceptives or the reliance on conventional estimation techniques (i.e., counting methods for ovulation detection) [63]. Although we used ovulation test kits to accurately detect the fertile phase, we did not collect other hormonal measures.

### Auditory sexually dimorphic

Our results support our hypothesis (H3), that during the fertile phase, participants would perceive the durations of briefly heard masculinized male voices as longer than femininized male voices, but not during the non-fertile phase. Our findings align with previous research concerning women’s cycle shift preference towards men’s voices [54–56]. This may be explained by the notion that during ovulation, women may aim to maximize their chances of conception by preferring men with better genetic traits (i.e., see [33] for a review). Since this cycle’s shift was not observed in the visual modality, one might reason that women’s heightened speech perception during ovulation could account for this difference (i.e., [104–106]).

On the other hand, our findings contrast with recent research which has found no evidence that cycle phase, conception risk, or steroid hormone levels affect women’s preferences for masculine voices [68]. Instead, the authors found an increased attraction to men’s voices during ovulation, regardless of how masculine they sounded. This general shift in women’s voice preferences is supported by additional studies suggesting that hormonal changes during the menstrual cycle influence overall sexual desire, but not a desire for uncommitted sexual relationships [107,108]. However, our results do not support these findings, as they did not indicate an overall overestimation of male voices during the fertile phase. Specifically, there were no significant differences in temporal reproduction between male voices and the control stimulus (600 Hz sine wave).

Although we cannot directly compare our results with those of previous studies on cyclical shifts in preferences for masculine voices, as our focus was on time perception rather than attractiveness perception per se, some distinctions should still be addressed. Prior studies recorded male participants attempting to attract a female [54,55], while in our study, participants heard just the vowel/i/. This difference is noteworthy, as the length and type of auditory stimuli are known to influence attractiveness perception. For instance, a study demonstrated that the perceived attractiveness of hearing the word “hello” was higher than that of hearing a single vowel [109]. However, other research recorded male voices reading an excerpt from a standard voice passage and found no evidence of a cycle shift in women’s preferences for masculinized voices [68]. Therefore, the content of the message (i.e., attempting to attract a female) and its duration may interact to influence perceived attractiveness, thereby affecting the perceived duration.

### Limitations and further studies

Some limitations of the present study should be mentioned. First, our sample was primarily comprised of relatively young Portuguese bisexual or heterosexual females. Additional research should explore the possible moderator role of the menstrual cycle in the effect of attractiveness on time perception across different cultural settings and age groups.

Also, the study of additional sexual orientations, such as homosexuality, may provide significant contributions to the underlying mechanisms regarding the influence of hormonal changes on cognitive processes. For example, testing whether homosexual women show differences in time reproductions during different phases of the cycle when viewing/hearing male or female faces/voices compared to heterosexual ones.

Additionally, by manipulating attractiveness/sexual dimorphism within blocks of trials but manipulating the type of stimuli/modality (visual attractiveness/visual dimorphic/auditory dimorphic) between blocks, we used the most convenient design to address our research questions. These questions centered on whether perceived attractiveness would influence time perception during ovulation. However, this design limits our ability to address other questions, such as whether the perceived duration between viewing attractive vs. masculinized male photos would differ for a given phase of the cycle.

Furthermore, future studies should explore a possible interaction effect between facial expressions and attractiveness on time perception. The influence of angry faces on time perception, which has been shown to increase duration estimates, is well established [13]. Moreover, the interaction between angry faces and attractiveness has been explored, suggesting an overestimation of angry attractive faces, but not of unattractive faces, compared to neutral ones [12]. However, there is no evidence of a possible interaction between happiness and attractiveness on time perception. Although the type of facial expressions was controlled for each set of stimuli (attractive/unattractive), emotions with positive valence may have enhanced the subjects’ arousal for the attractive male models but not unattractive photos [110]. Therefore, a ceiling effect on the preference for attractive faces might have occurred, and this should be investigated in future studies.

In the present study, voices were stretched or compressed to achieve the desired time interval ranges, which respectively lowers or raises the pitch. This adjustment was made before pitch manipulation to ensure objective differences between masculinized and feminized male voices. However, future research should investigate whether using more natural stimuli, such as reading text excerpts of varying lengths and collecting testosterone measurements from male vocal donors, might influence women’s time perception of male voices. The confounding of sexual dimorphism and attractiveness in the stimuli used in this study limits the ability to isolate the specific effect of masculinity on time perception of male voices, particularly in the context of the menstrual cycle. Future research could address this limitation by creating stimuli that are equally attractive but vary in dimorphism, or equally dimorphic but differ in attractiveness. This would help clarify whether masculinity has a unique effect on time perception of male voices across different menstrual cycle phases or if the effect is dependent on attractiveness.

Additionally, further research should explore whether differences in time perception emerge in cross-modal scenarios by presenting videos that combine attractive visual and auditory features of males. Moreover, the research field of attractiveness time perception could be extended to other sensory modalities. For example, olfactory cues have been linked to influence both visual attractiveness [111], and time perception [112]. Furthermore, the menstrual cycle may play an important role in these effects, as positive associations between body odor and attractiveness were found only when female odor raters were in the most fertile phase of their menstrual cycle [113,114].

Another important question for future research is whether results would vary for other phases of the hormonal cycle, for example, pre-menstrual, mid-luteal, and mid-follicular. Our design only compared the ovulatory and menstrual phases, which may not fully capture the hormonal variation throughout the cycle. Estradiol levels are significantly higher during the ovulation phase than in the menstrual phase, while progesterone levels remain relatively low in both phases. Although evidence suggests that changes in estradiol during the cycle predict shifts in preferences for masculinity cues [56,59], other researchers have not found support for this effect [102,115]. An alternative explanation is that preferences for femininity might be strongest when progesterone levels are increased [116]. Additionally, there is some concern that women’s awareness of their menstrual status could influence the results [73,117,118], rather than hormonal changes per se. Therefore, further studies should collect salivary samples to compare hormonal differences (e.g., estradiol, and progesterone) within participants across different phases of the cycle.

Finally, we suggest that future studies try replicating our findings using different time perception tasks, such as temporal bisection or verbal estimation tasks. When replicating the present work, it could be interesting to include some psychophysiological measures, like eye-tracking, EEG, skin conductance response (SCR), and heart rate (HR), to gain additional insights on the additive or multiplicative influence of arousal and attention on time perception. Moreover, researchers have shown that more attractive women will show stronger preferences for both facial [119] and vocal masculinity in men [120]. Hence, future studies should explore whether the attractiveness of participants has an impact on the observed results.

## Conclusions

The results of the present study indicate that hormonal changes associated with the menstrual cycle modulate women’s auditory perception of sexually dimorphic male voices. Longer time estimates were observed for masculinized male voices during the fertile phase but not during menstruation. Additionally, we replicated previous findings regarding the dilation effect of attractive faces on time perception. However, no differences in time estimations were found between masculine and feminine male faces. Future research could build on these findings by exploring alternative methodologies, such as collecting hormonal salivary samples and extending data collection to other phases of the menstrual cycle. Overall, our findings align with the literature on the acceleration of the internal clock related to attractiveness perception. Lastly, this study provides initial evidence for an adaptive time perception mechanism influenced by the menstrual cycle.

### Declaration of generative AI and AI-assisted technologies in the writing process

During the preparation of this work the authors used Grammarly and ChatGPT 4.o in order to improve the language of the manuscript. After using these tools, the authors reviewed and edited the content as needed and take full responsibility for the content of the publication.

## Supporting information

S1 Fig Reproduced durations for the visual attractiveness block across all conditions, for both the ovulation phase (left grid) and menstruation phase (right grid) . Error bars represent the standard error of the mean.
(TIF)

S2 Fig Reproduced durations for the visual dimorphic block across all conditions, for both the ovulation phase (left grid) and menstruation phase (right grid) . Error bars represent the standard error of the mean.
(TIF)

S3 Fig Reproduced durations for the auditory dimorphic block across all conditions, for both the ovulation phase (left grid) and menstruation phase (right grid) . Error bars represent the standard error of the mean.
(TIF)

S1 Table Table showing socio-demographic variables. Results are shown in absolute and relative (%) frequencies.
(DOCX)

S1 File Feminized male. (TIF)

S2 File Masculinized male. (TIF)
