# Supplementary figures and images for: Time perception of attractive male faces and voices: The role of women’s menstrual cycle
Source: PLoS One. 2025 Apr 24;20(4):e0321956. doi: 10.1371/journal.pone.0321956 (PMC12021210; doi:10.1371/journal.pone.0321956)

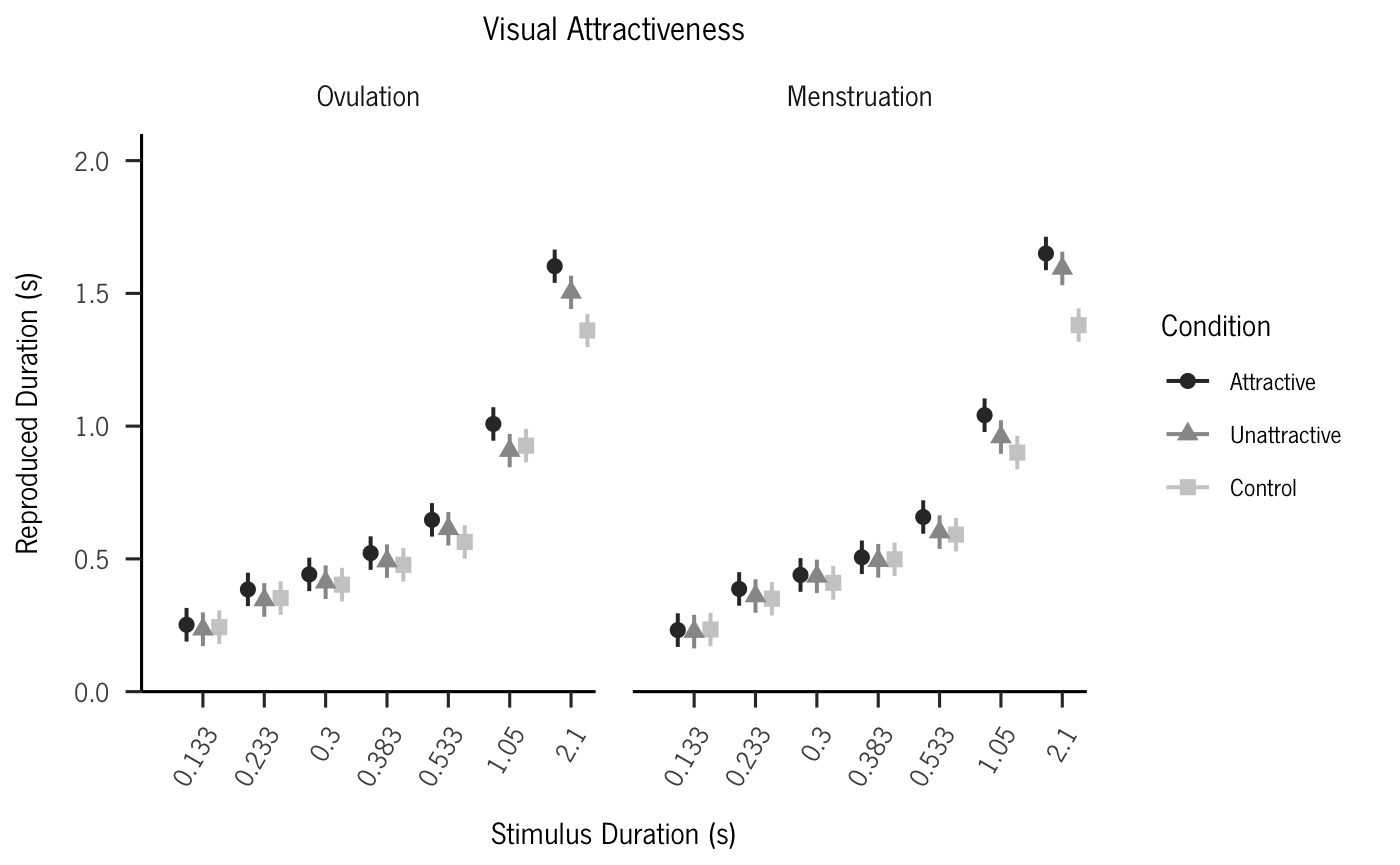

Supplement: S1 Fig — . Error bars represent the standard error of the mean. (TIF) [file pone.0321956.s001.tif]

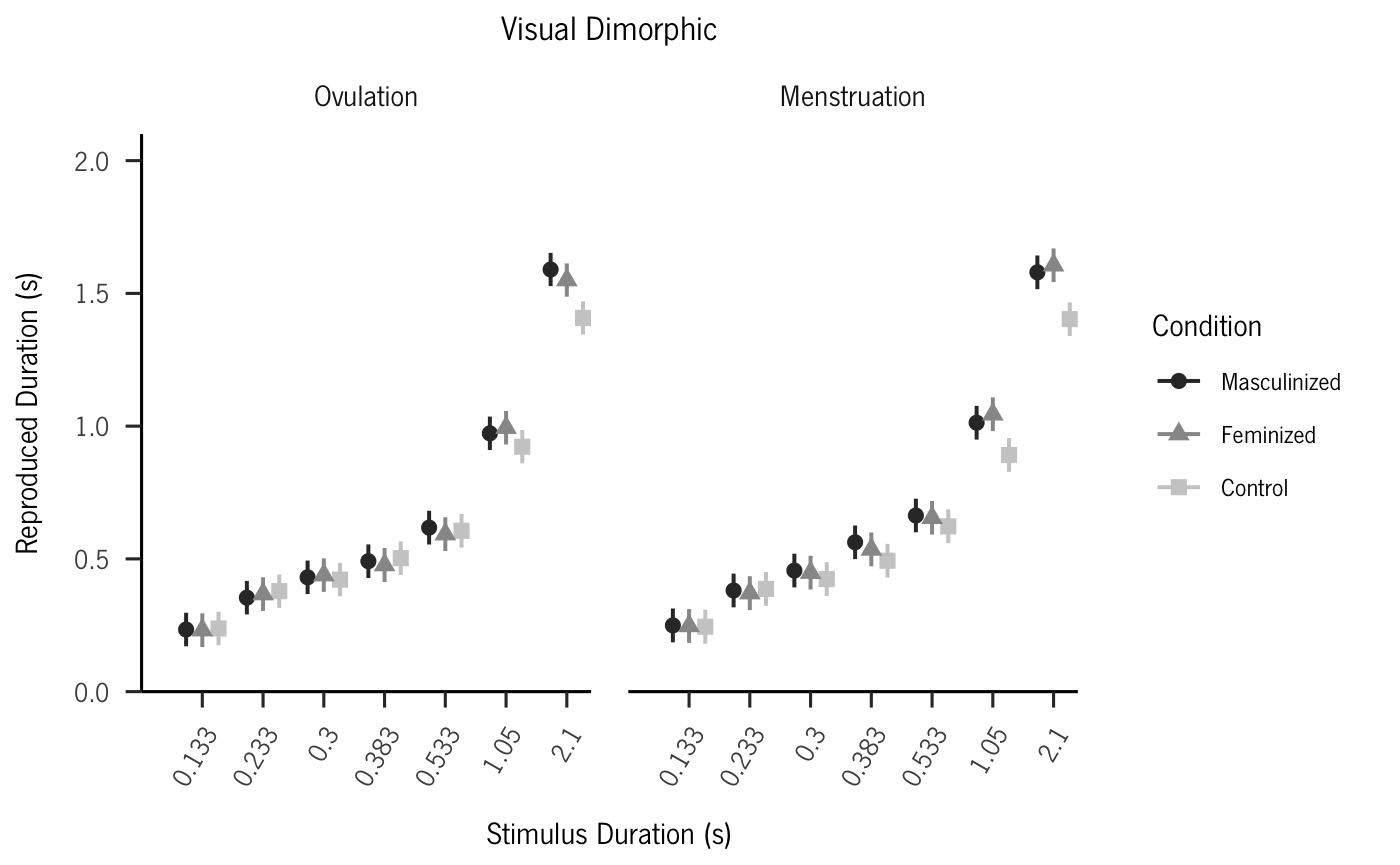

Supplement: S2 Fig — . Error bars represent the standard error of the mean. (TIF) [file pone.0321956.s002.tif]

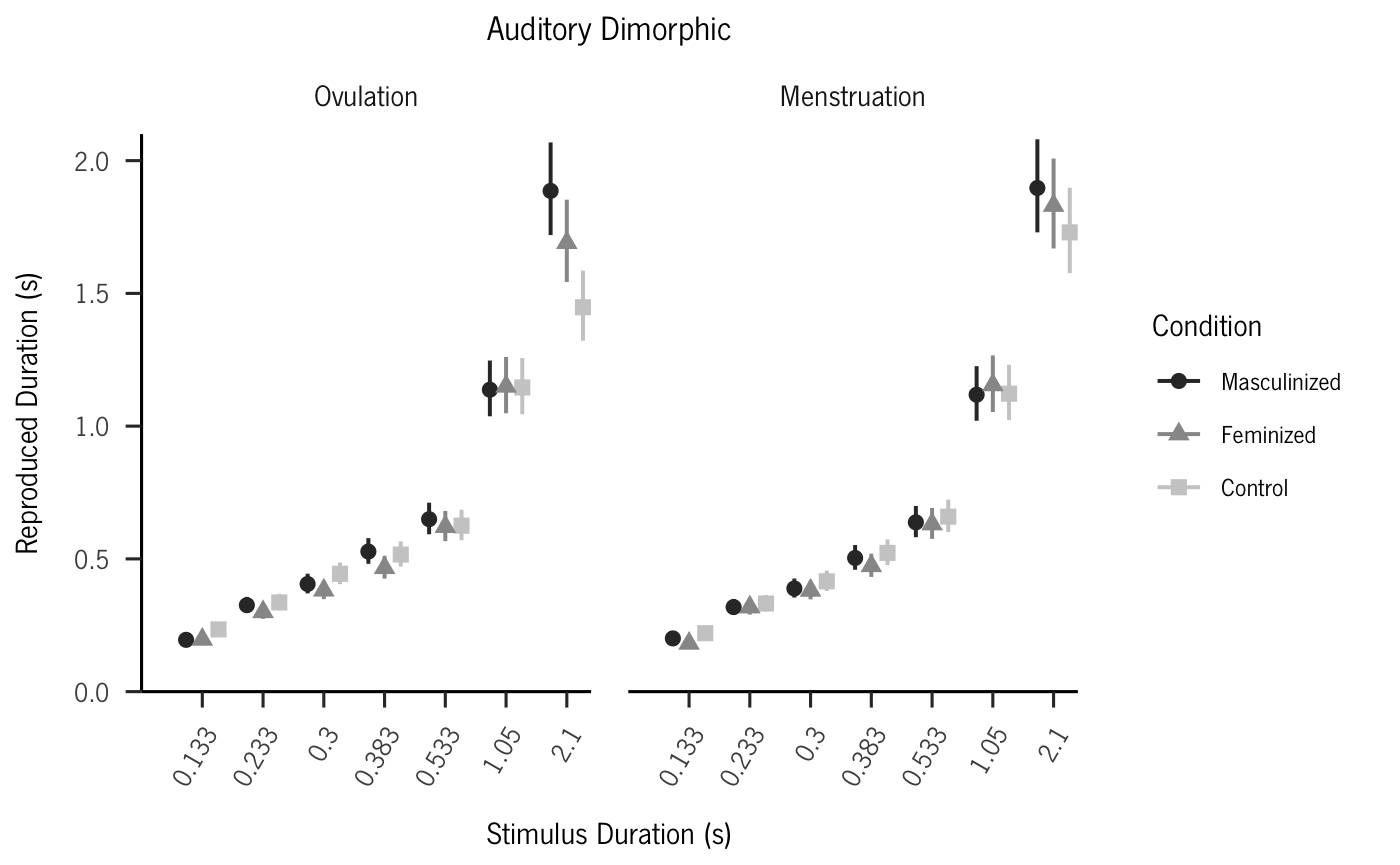

Supplement: S3 Fig — . Error bars represent the standard error of the mean. (TIF) [file pone.0321956.s003.tif]

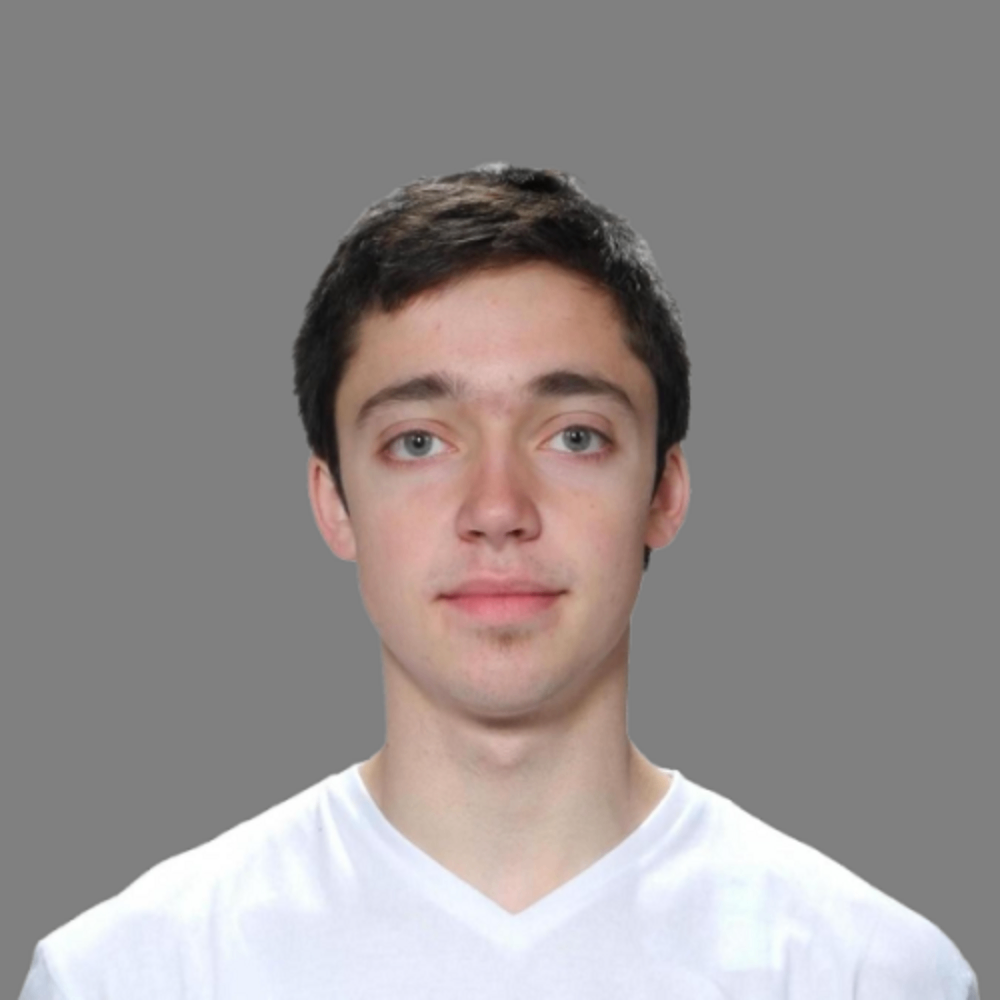

Supplement: S1 File — (TIF) [file pone.0321956.s005.tif]

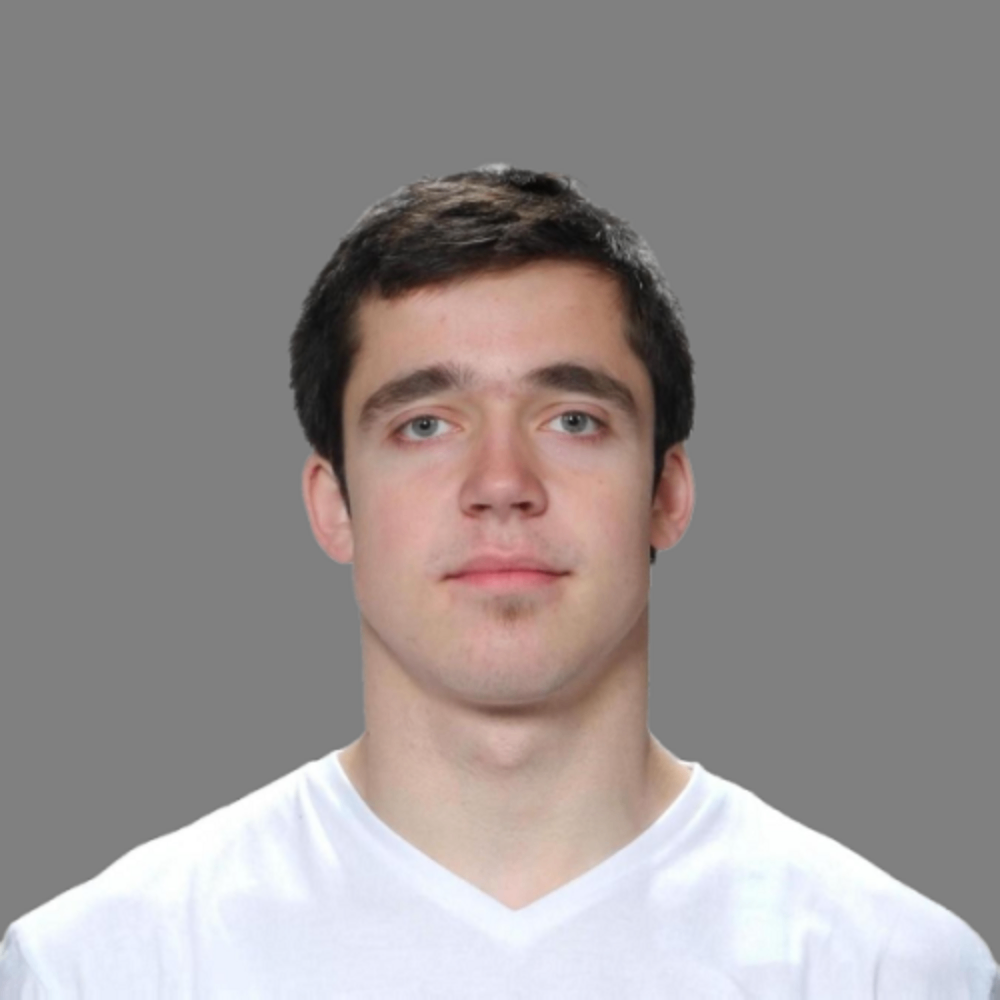

Supplement: S2 File — (TIF) [file pone.0321956.s006.tif]
