# Supplementary material for: Time perception of attractive male faces and voices: The role of women’s menstrual cycle
Source: PLoS One. 2025 Apr 24;20(4):e0321956. doi: 10.1371/journal.pone.0321956 (PMC12021210; doi:10.1371/journal.pone.0321956)
Supplement: S1 Table — Results are shown in absolute and relative (%) frequencies. (DOCX) [file pone.0321956.s004.docx]

| Personal characteristics | Level | n (%) |
| --- | --- | --- |
| Nationality | Portuguese | 40 (83.3) |
|  | Brazilian | 7 (14.6) |
|  | Spanish | 1 (2.1) |
| Relationship Status | Single | 30 (62.5) |
|  | Relationship | 18 (37.5) |
| Socioeconomic status | Low  Medium-Low | 1 (2.1)  9 (18.75) |
|  | Medium  Medium-High | 22 (45.8)  7 (14.6) |
|  | High  Non-disclosure | 1 (2.1)  8 (16.7) |
| Employment status | Employed | 2 (4.2) |
|  | Student | 43 (89.6) |
|  | Working student | 3 (6.3) |
